# Systematic analysis of the sugar accumulation mechanism in sucrose- and hexose- accumulating cherry tomato fruits

**DOI:** 10.1186/s12870-022-03685-8

**Published:** 2022-06-22

**Authors:** Lulu Sun, Jianli Wang, Liqiang Lian, Jian Song, Xueni Du, Wenke Liu, Wenchao Zhao, Liu Yang, Changbao Li, Yong Qin, Rui Yang

**Affiliations:** 1 Beijing Key Laboratory for Agricultural Application and New Technique, Beijing University of Agriculture, No.7 Beinong Road, Beijing, 102206 Changping District China; 2 College of Plant Science and Technology, Beijing University of Agriculture, No.7 Beinong Road, Beijing, 102206 Changping District China; 3 Beijing Vegetable Research Centre, Beijing Academy of Agriculture and Forestry Sciences, Beijing, 100097 Haidian District China; 4 Department of Forestry and Horticulture, Xinjiang Agricultural University, No.311 Nongda Dong Road, Urumqi, 830052 Xinjiang China

**Keywords:** Sucrose, Hexose, Accumulation, Cherry tomato, Phloem unloading

## Abstract

**Background:**

Sugar content is an important indicator of fruit quality. Except for a few wild tomato species that accumulate sucrose in the fruits, most cultivated tomato species accumulate hexose. Although several studies have focused on wild sucrose-accumulating tomato, the sucrose accumulation mechanism is still unclear.

**Results:**

Here, two homozygous inbred cherry tomato lines (‘TB0023’ and ‘TB0278’, which accumulated sucrose and hexose, respectively) were selected to analyze the sugar accumulation mechanism. Carbohydrate analysis, cytological observation, gene expression and enzyme activity analysis and proteomics methods were used in this study. The results indicated that glucose and fructose were absolutely dominant in the soluble sugar content of hexose-accumulating cherry tomato fruit, while sucrose and a certain proportion of hexose were the main forms of soluble sugar in sucrose-accumulating cherry tomato fruit. The phloem unloading pathway of the hexose-accumulating cherry tomato fruit switched from symplastic to apoplastic during fruit development, and the sucrose-accumulating cherry tomato probably had a mixed unloading pathway involving the symplastic and apoplastic. High activity of acid invertase (AI), sucrose phosphate synthase (SPS), sucrose synthase (SS) and sugar transporters LeSUT1, SlSWEET2a and SlSWEET12c were important factors for hexose accumulation in the hexose-accumulating cherry tomato fruit, while LeSUT2, SPS, SS, SlSWEET1b, SlSWEET5b, SlSWEET11b, SlSWEET7a, SlSWEET14 were responsible for solute sugar accumulation in the sucrose-accumulating cherry tomato.

**Conclusions:**

This study provides detailed evidence for elucidation of the tomato sugar accumulation mechanism from the perspective of cell structure, physiology and molecular biology, providing a theoretical basis for the improvement of tomato quality and aiding the utilization of tomato genetic resources.

**Supplementary Information:**

The online version contains supplementary material available at 10.1186/s12870-022-03685-8.

## Background

Tomato has high economic value and is widely consumed, and the functional value of the fruit is primarily determined by the levels of sugar, lycopene, vitamin C and polyphenols [1], which are beneficial to human health [2, 3]. Sugar content is the key component that affects tomato quality and customer preference and is important for various aspects of plant development, for example, sugars provide energy and carbon structural elements for plant growth and function as signaling molecules in many developmental processes [4, 5].

Plant photosynthesis produces sucrose, which is transported long-distance along the phloem to sink organs, such as seeds, roots, and young leaves. Then this sucrose is unloaded from the phloem to the sink cells though the symplastic pathway (via plasmodesmata) or apoplastic pathway (via sugar transporters), where it can be used as a nutrient for the growth and development of the sink cells or being directly stored as an energy source [6]. The accumulation of sugar in the sink organs depends mainly on the input of sugar and biochemical processes such as sugar synthesis and decomposition [7].

Different forms of sugar accumulation in tomato fruits has been observed. Most tomato cultivars mainly accumulate hexoses, accumulating sucrose at low levels [8, 9]. However, some wild relative species, such as *Lycopersicon hirsutum*, *Lycopersicon chmielewskii* and *Lycopersicon peruvianum* [9], and a few cultivated tomato varieties [10] accumulate higher levels of sucrose than typical hexose-accumulating tomato plants, and in the final developmental stages, the fruit sugar levels of these genotypes are approximately 10 times that of ordinary tomatoes and primarily include sucrose, whereas the levels of glucose and fructose are relatively low [9]. Interestingly, genotypes that accumulate high levels of total sugars also accumulate high levels of sucrose, while fruits of genotypes that accumulate low levels of sugars do not accumulate sucrose [9, 11]. Diversity of sugar accumulation among different tomato species and cultivars reflects differences in sugar metabolism during fruit development, which could be regulated to create more valuable tomato varieties [12, 13].

However, only a few studies have focused on wild tomato species that accumulate sucrose, and little information exists on other tomato species. Previous studies on *L. peruvianum* and *L. esculentum* reported that the increase in sucrose levels in sucrose-accumulating fruits was associated with invertase and sucrose phosphate synthase (SPS) activities. SPS seems to play a key biochemical role in the accumulation of sucrose and the establishment of high sugar content in tomato fruits [14, 15]. A study on *L. chmielewskii*, a wild tomato species that accumulates high levels of sucrose in its mature fruits, found that the lack of acid invertase (AI) activity in sucrose-accumulating fruit was the only significant enzymatic difference between the sucrose-accumulating and hexose-accumulating fruit, whereas SPS did not play an important role [16]. Another report noted that the accumulation of hexose in cultivated tomato may be related to the accumulation of starch before fruit ripening, but the accumulation of sucrose in *Lycopersicon cheesmanii* may be related to the continuous input of sucrose in the later stage [9]. Collectively, these studies did not thoroughly analyze the mechanism of sucrose and hexose accumulation.

The role of sugar transporters has rarely been mentioned in these previous studies, but sucrose transporter (SUT), sugars will eventually be exported transporter (SWEET) proteins, etc. have been shown to play important roles in tomato fruit sugar accumulation. Antisense inhibition of *LeSUT1* and *LeSUT2* reduced the fertility of fruits [17], SlSWEET7a, SlSWEET14 [18], SlSWEET15[19] and a Fgr^H^ allele [20] had been shown to affect carbohydrate allocation in tomato fruits. Recently, we identified and developed a sucrose-accumulating cherry tomato cultivar ‘TB0023’ that has not previously been reported, and another homozygous inbred cherry tomato cultivar ‘TB0278’ that accumulated hexose was used as a control to analyze the sugar accumulation mechanism of tomato fruit. In this study, we compared the fruit quality and sugar content of sucrose-accumulating and hexose-accumulating cherry tomato plants. Moreover, we systematically investigated the phloem assimilate unloading pathway and sugar accumulation mechanism by cytological observation, qRT-PCR, enzyme activity determination and proteomics analysis. These results provide a better understanding of the accumulation of different types of sugar in tomato fruits.

## Results

### Growth and quality identification

Upon comparing the growth of the sucrose-accumulating cherry tomato (S) and hexose-accumulating cherry tomato (H) lines, we found that apart from the number of flowers, there was no significant difference between the two lines, including in plant height, number of leaves, and number of fruits (Fig. S1).

To study the development of tomato fruit in detail, the fruits of the two cherry tomato lines were classified into 5 stages (I, immature; II, mature; III, green breaker; IV, pink; V, red ripe) according to the tomato growth period standards developed by the United States Department of Agriculture (USDA) and the growth curves of the fruits of the two cherry tomato genotypes (Fig. S2).

**Fig. 1:**
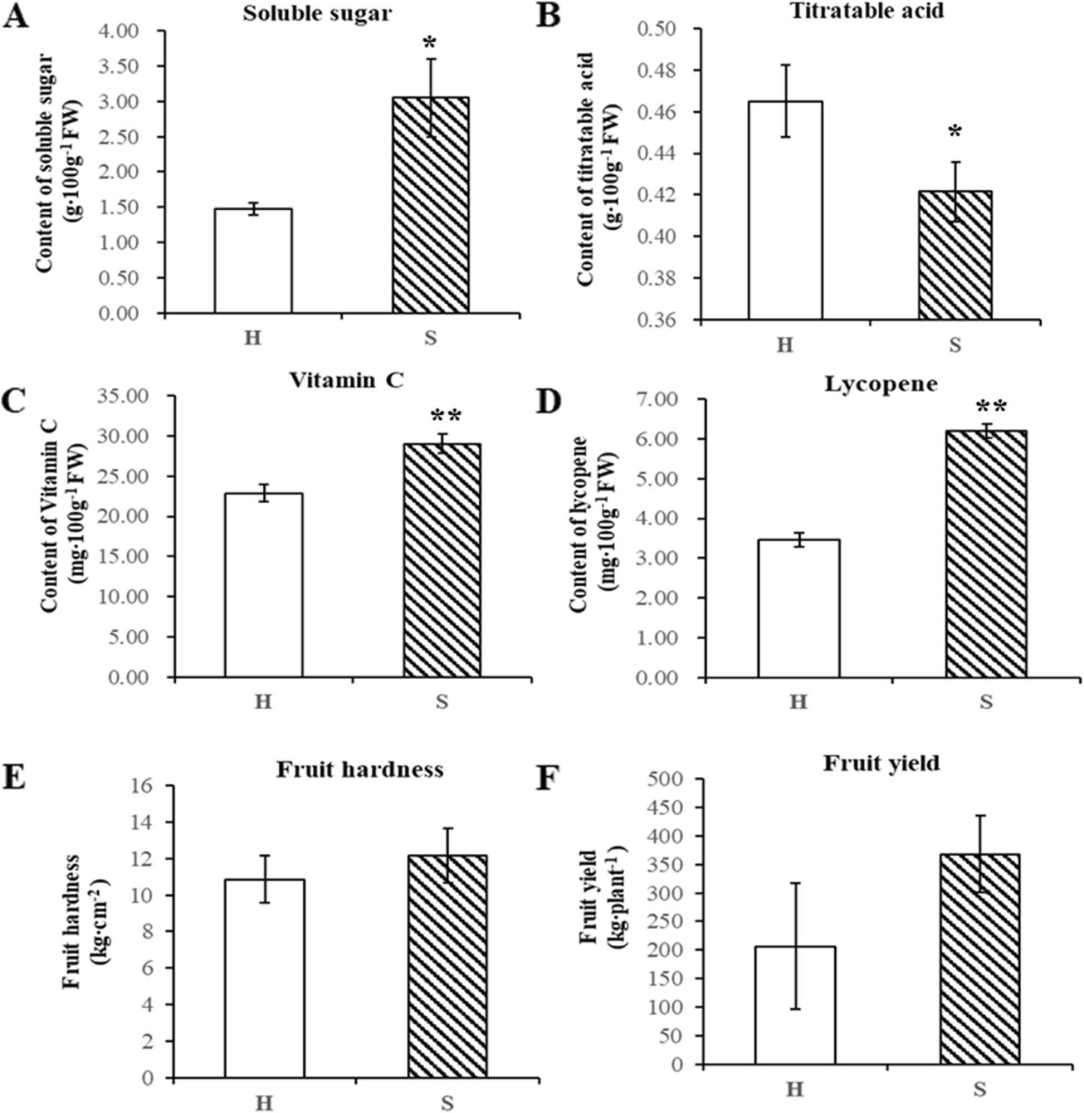
Analysis of the fruit quality and yield of tomato genotypes with different sugar accumulation levels at the red ripe stage (stage V). **A** Soluble sugar content. **B** Titratable acid content. **C** Vitamin C content. **D** Lycopene content. **E** Fruit hardness. **F** Fruit yield. Student’s t test, *P < 0.05, **P < 0.01. Abbreviations: H, hexose-accumulating cherry tomato; S, sucrose-accumulating cherry tomato

The analysis of the fruit quality and yield of the two cherry tomato lines indicated that compared with the H-type cherry tomato, the S-type cherry tomato fruit had higher soluble sugar, vitamin C and lycopene levels and lower organic acid levels at the red ripe (V) stage. Moreover, there was no significant difference in fruit firmness and yield (Fig. 1). Therefore, the quality of the S-type cherry tomato was better than that of H-type, which is helpful for the genetic application of sucrose-accumulating tomato materials.

### Carbohydrate accumulation analysis

**Fig. 2:**
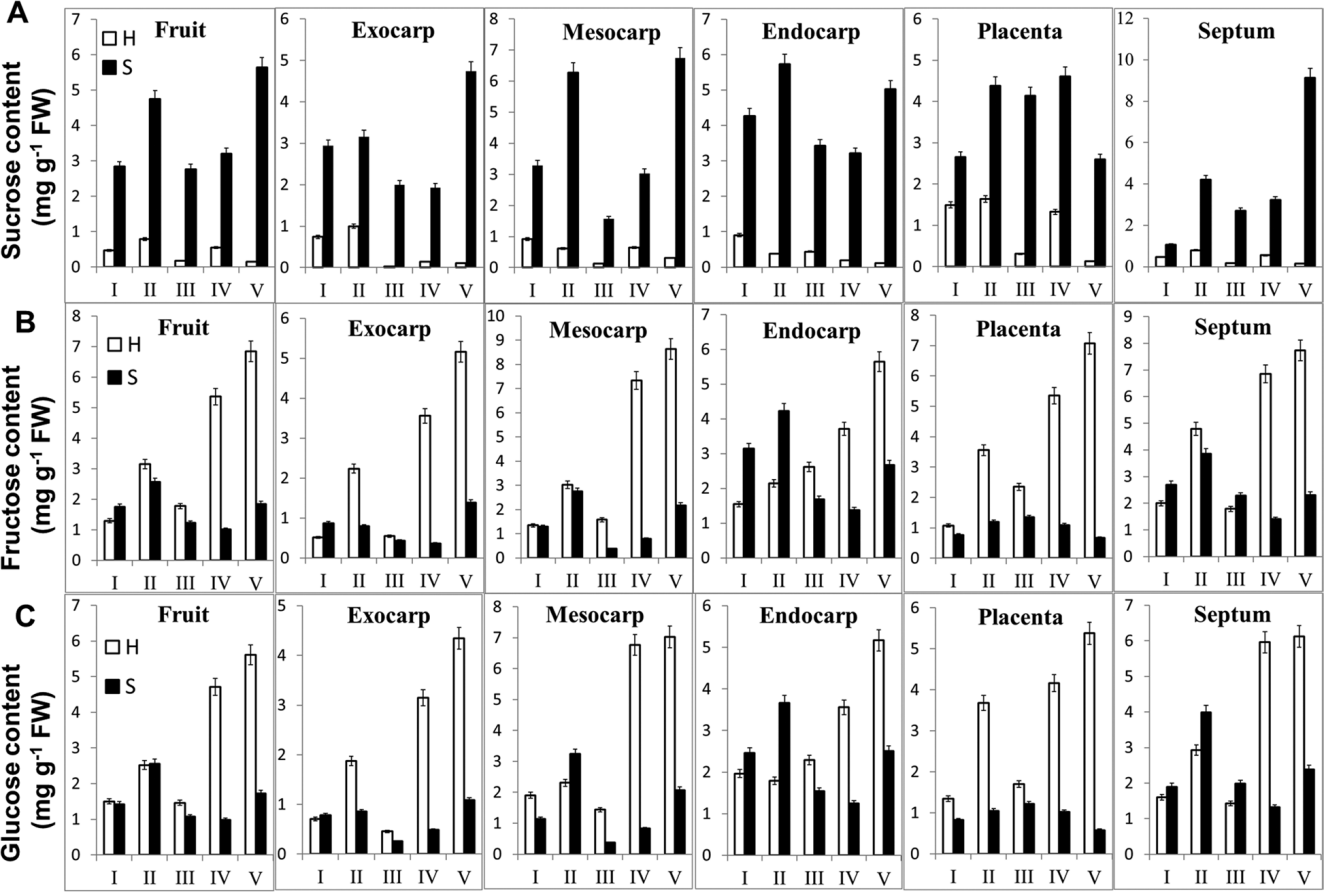
Carbohydrate content in different tissues and stages of two cherry tomato fruit lines. **A** Sucrose content. **B** Glucose content. **C** Fructose content. Data are expressed as the mean ± SEM (*n* = 3). Student’s t test, *P < *0.05*; **P < *0.01*. Abbreviations: H, hexose-accumulating cherry tomato; S, sucrose-accumulating cherry tomato; I, immature stage; II, mature green stage; III, breaker stage; IV, pink stage; V, red ripe stage

The levels of sucrose, fructose and glucose at different stages (Stages I to V) and parts (whole fruit, exocarp, mesocarp, endocarp, placenta and septum) of fruits of the sucrose-accumulating and hexose-accumulating cherry tomato lines were analyzed (Fig. 2). Regardless of stage and fruit part, the S-type cherry tomato accumulated more sucrose than the H-type, while the accumulation of glucose, fructose and starch in the H-type cherry tomato was significantly higher than that in the S-type. It is worth mentioning that hexose had an absolute predominance in the H-type cherry tomato, while the sucrose content was very low in this line. However, in addition to sucrose, hexose also constituted a certain proportion (approximately 40%) of the total soluble sugar content, in the S-type cherry tomato, especially in the endocarp. Sucrose accumulation in whole fruit and different fruit tissues of the S-type tomato showed a similar trend; the sucrose content peaked at the mature stage (stage II) first, then decreased and then peaked again at the red ripe stage (stage V). The accumulation of glucose and fructose in the H-type tomato fruit presented a similar trend as that of sucrose in the S-type cherry tomato and was high mainly in the later stages.

### Cytological pathway of phloem unloading of the two cherry tomato lines

**Fig. 3:**
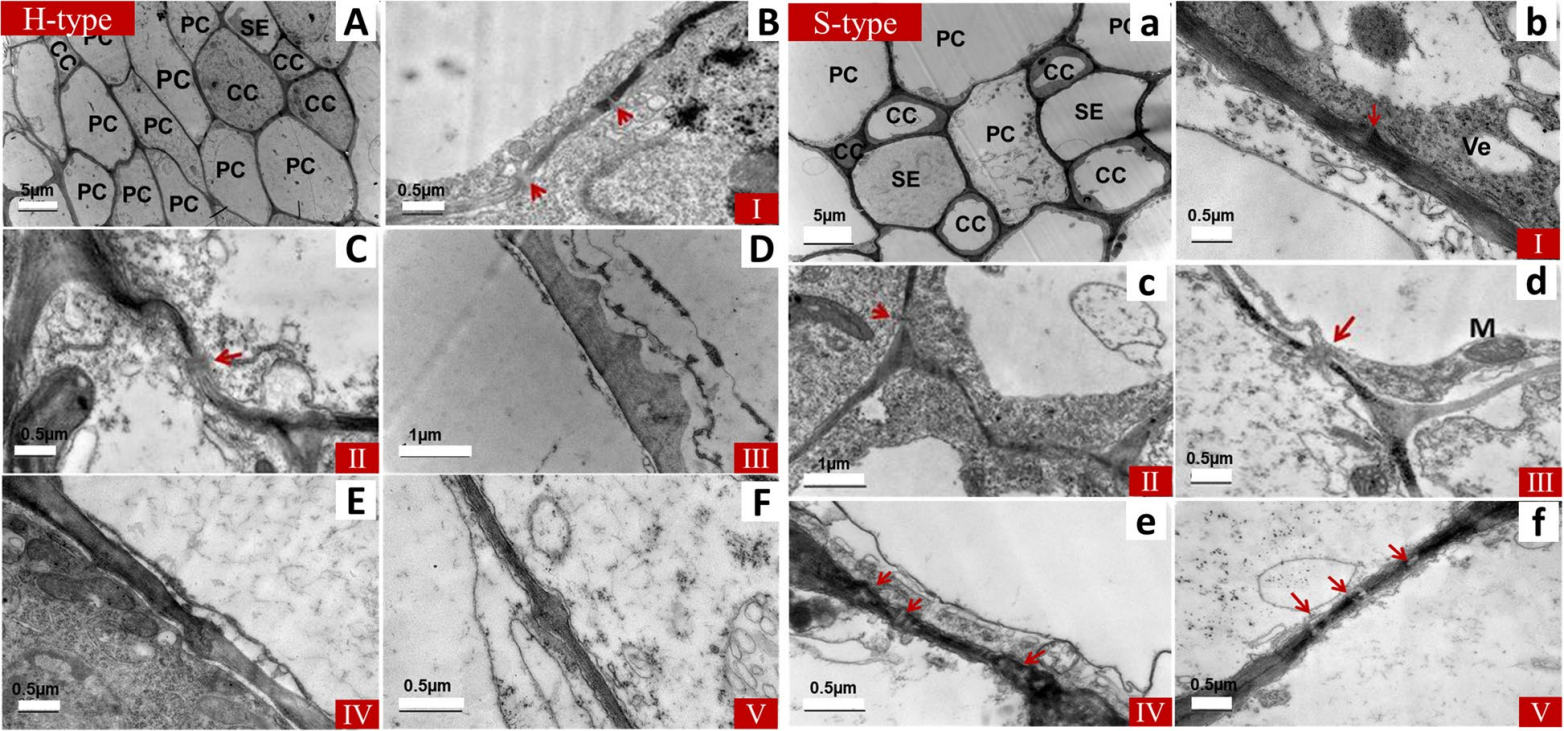
Ultrastructure of the sieve element-companion cell complex (SE/CC) and its surrounding parenchyma cells (PCs) in developing tomato fruit. All sections were cut transversely. Left, hexose-accumulating cherry tomato. Right, sucrose-accumulating cherry tomato. The structures of the phloem of the two kinds of cherry tomato are shown in the upper left picture. The SEs, CCs and PCs are labeled. I-V, ultrastructure between SE/CCs and PCs during developmental stages I to V. plasmodesmata are labeled by red arrows. Abbreviations: SE, sieve element; CC, companion cell; PC, phloem parenchyma cell; PD, plasmodesmata; M, mitochondrion; Ve, vacuole; H, hexose-accumulating cherry tomato; S, sucrose-accumulating cherry tomato. I, immature stage; II, mature green stage; III, breaker stage; IV, pink stage; V, red ripe stage

The type of sugar accumulation in the fruit should be related to the phloem unloading pathway of tomato fruit. To investigate the cytological basis of the accumulation of sugar during fruit development and ripening, we examined the connection between the phloem and surrounding cells. Here, hexose-accumulating cherry tomato was observed as a control, and previous research reported that the phloem unloading pathway of tomato fruit shifted from being symplastic at an early stage to apoplastic pathway for rapid hexose accumulation at a later stage [21]. Observation of the plasmodesmata between the phloem and surrounding cells by transmission electron microscopy (TEM) proved the previous conclusion. There were many plasmodesmata at early stages (stage I to stage III) and few plasmodesmata at later stages (stage IV to stage V) between the sieve element/companion cell complexes (SE/CCs) and parenchyma cells (PCs) in the H cherry tomato fruit (Fig. 3; Table 1). However, in the S-type cherry tomato fruit, plasmodesmata between the SE/CCs and PCs existed throughout the fruit development period (stage I to stage V) and their number decreased slightly at later stages. Of course, there was also a sharp increase in stage II, indicating strong symplastic transport (Fig. 3; Table 1). The results indicated that the phloem unloading pathway of the S-type cherry tomato was probably a mixed unloading pathway involving symplastic and apoplastic, and that the symplastic pathway was dominant at the early stage of fruit development.

**Table 1:** Plasmodesmal densities (PDDs) between different cells of the phloem during fruit developmental stages I to V

Genotype	Developmental stages	SE/CC	SE/PC	CC/PC	PC/PC
S	I	0.90 ± 0.10	0.10 ± 0.02	0.26 ± 0.04	0.28 ± 0.06
	II	0.66 ± 0.06	0.26 ± 0.04	0.92 ± 0.13	0.82 ± 0.51
	III	0.60 ± 0.08	0.28 ± 0.04	0.20 ± 0.05	0.24 ± 0.06
	IV	0.75 ± 0.11	0.20 ± 0.05	0.12 ± 0.03	1.05 ± 0.25
	V	0.84 ± 0.10	0.17 ± 0.02	0.10 ± 0.02	1.62 ± 0.07
H	I	0.93 ± 0.11	0.14 ± 0.01	0.69 ± 0.20	0.68 ± 0.34
	II	0.64 ± 0.07	0.08 ± 0.02	0.45 ± 0.08	0.61 ± 0.01
	III	0.48 ± 0.07	0.18 ± 0.04	0.78 ± 0.08	0.57 ± 0.13
	IV	0.95 ± 0.13	0.03 ± 0.01	0.00 ± 0.00	0.53 ± 0.06
	V	0.78 ± 0.09	0.03 ± 0.01	0.04 ± 0.02	0.51 ± 0.17

Units of PDD, number of plasmodesmata μm-1 cell length. Each value is the mean ± standard deviation of 30 replicates for PDD. Abbreviations: *SE* sieve element, *CC* companion cell, *PC* phloem parenchyma cell, *PDD* plasmodesmal density, *H* hexose-accumulating cherry tomato, *S* sucrose-accumulating cherry tomato

### Proteomics analysis

**Fig. 4:**
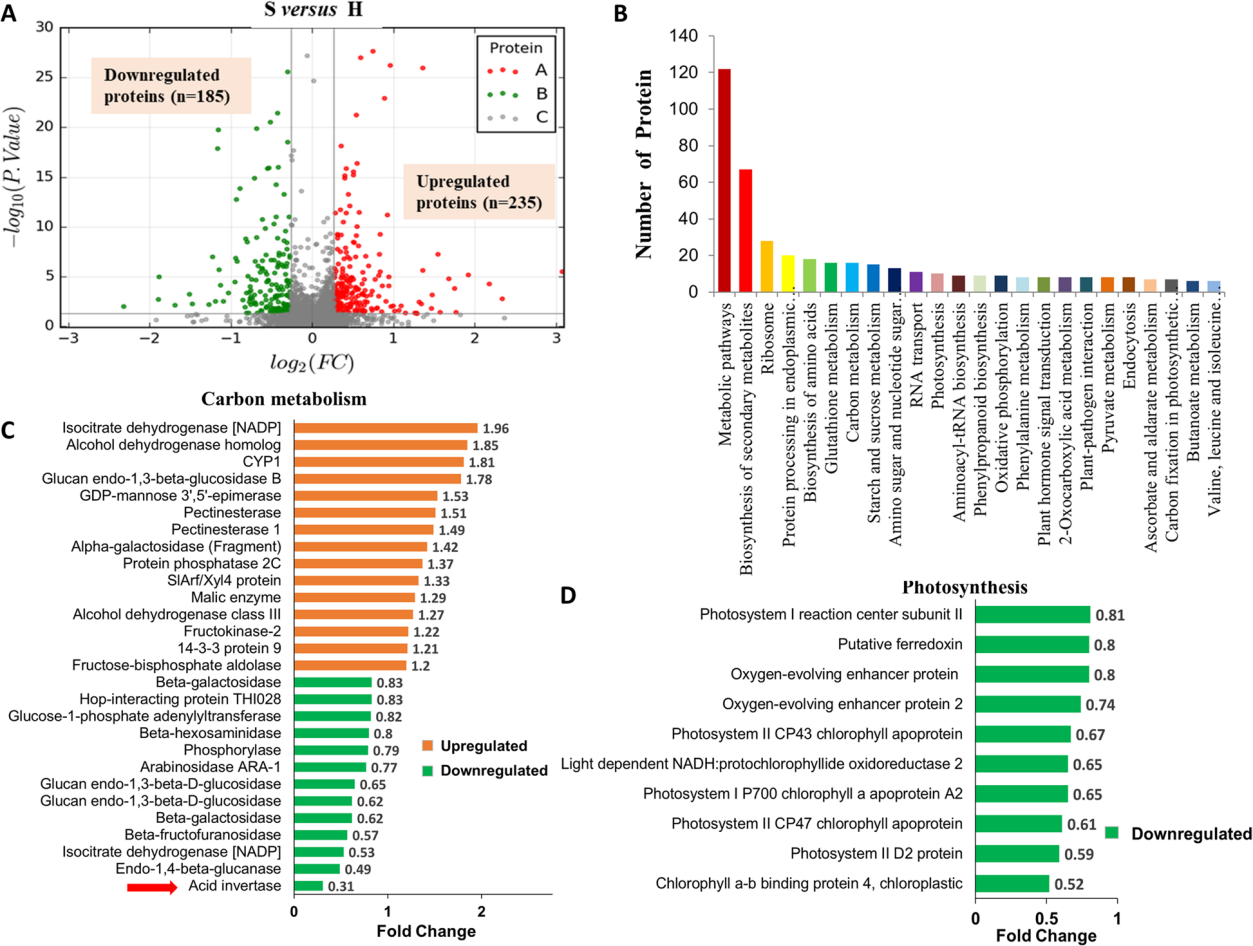
Proteomics analysis of ripe fruits of two types of tomato using iTRAQ technology. A, Volcano plot of downregulated and upregulated proteins based on proteomics data from the sucrose-accumulating tomato genotype versus the hexose-accumulating tomato genotype. B, Functional categories of differentially expressed proteins in proteomics data identified by COG. C, Differentially expressed proteins involved in carbohydrate metabolism. D, Differentially expressed proteins involved in photosynthesis. Abbreviations: iTRAQ, isobaric tags for relative and absolute quantitation; H, hexose-accumulating cherry tomato; S, sucrose-accumulating cherry tomato; FC, fold change

To further analyze the mechanism of sugar accumulation in the S- and H-type cherry tomato plants, the iTRAQ method was applied for proteome sequencing of ripe fruits of the two types of tomato (Fig. S3). Proteomics analysis indicated that there were 420 differentially expressed proteins between the S- and H-type tomato ripe fruit, 235 of these proteins were upregulated, and 185 were downregulated (Fig. 4A).

COG analysis indicated that these differentially expressed proteins were mainly related to metabolic pathways, biosynthesis of secondary metabolism, biosynthesis of amino acids, carbon metabolism, starch and sucrose metabolism, sugar metabolism, and photosynthesis (Fig. 4B). Further KEGG analysis indicated that in sucrose-accumulating tomato, most of the differentially expressed proteins involved in starch and sucrose metabolism and photosynthesis were downregulated, while most of the differentially expressed proteins involved in fatty acid degradation were significantly upregulated (Fig. S4 and S5). These results showed that in addition to sugar metabolism, other metabolic pathways also changed accordingly with the different types of sugar accumulation between the two types of tomato. Differentially expressed proteins involved in carbon metabolism and photosynthesis are shown in Fig. 4C and D in detail. It is worth noting that AI was significantly downregulated in the sucrose-accumulating tomato, which was consistent with the results of the enzyme activity assay.

### Expression analysis of sugar transporters and related metabolic enzymes

To further analyze the sugar accumulation mechanism in tomato fruits, the transcript levels of three *SUT* genes and eight highly expressed *SWEET* genes in fruit [22], as well as the activities of several enzymes related to sugar metabolism were studied.

**Fig. 5:**
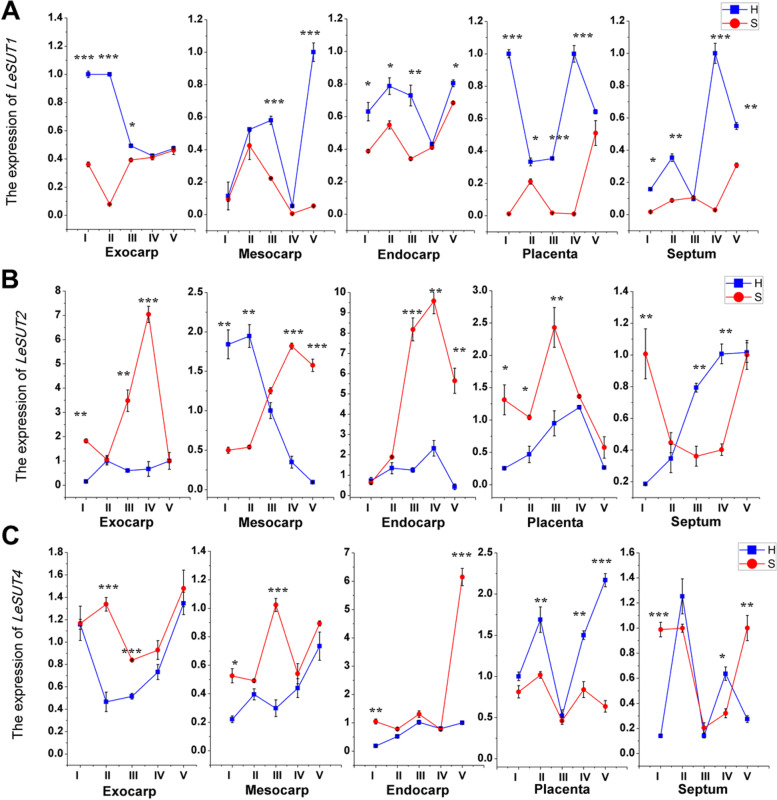
The mRNA levels of sucrose transporters (SUTs) in different tissues and developmental stages of tomato fruits of different sugar-accumulating genotypes. Error bars =  ± SEM (n = 3). Student’s t test, *P < *0.05*; **P < *0.01*; ***P < *0.001*. Abbreviations: H, hexose-accumulating tomato genotype; S, sucrose-accumulating tomato genotype; I, immature stage; II, mature green stage; III, breaker stage; IV, pink stage; V, red ripe stage

The mRNA expression levels of *SUT* genes in the two cherry tomato lines showed no obvious patterns during fruit development (Fig. 5). Overall, the H-type tomato had higher expression of *LeSUT1*, while the S-type tomato had higher levels of transcripts of *LeSUT2*, which indicates that the main SUT proteins responsible for sucrose transport in different sugar-accumulating cherry tomatoes may be different.

**Fig. 6:**
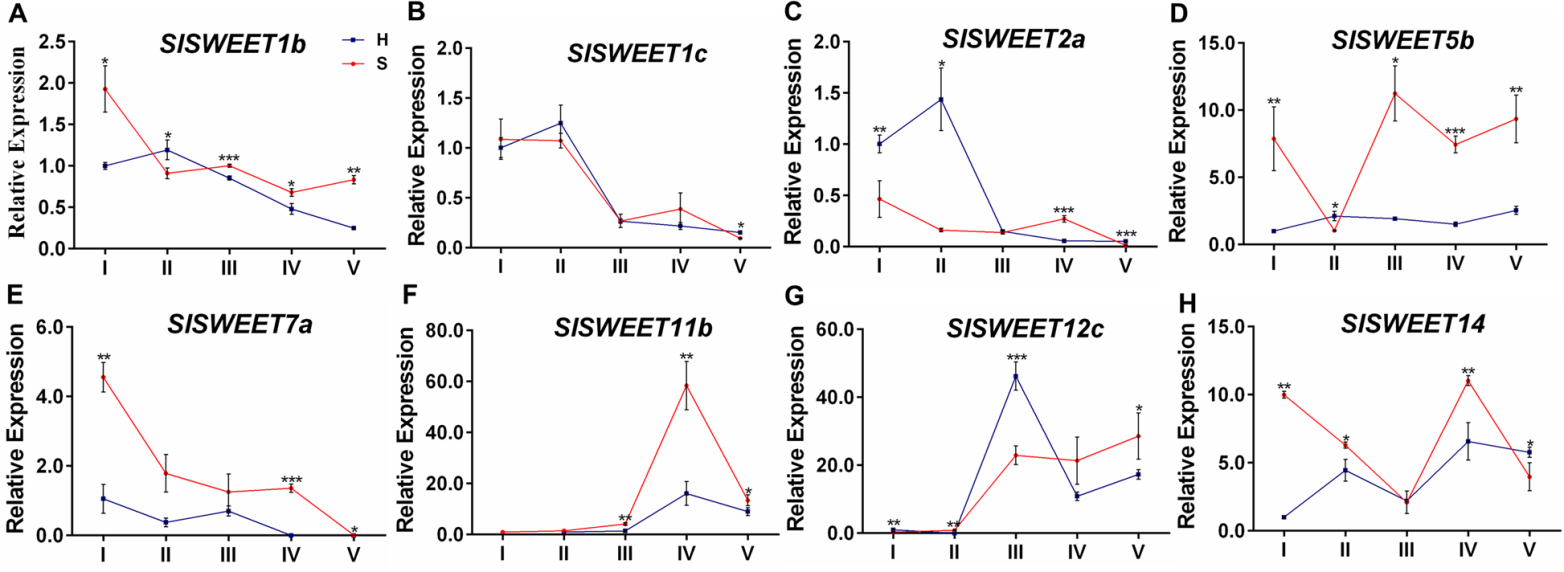
The mRNA levels of *SlSWEETs* in different developmental stages of tomato fruits of different sugar-accumulating genotypes. Error bars =  ± SEM (n = 3). Student’s t test, *P < *0.05*; **P < *0.01*; ***P < *0.001*. Abbreviations: H, hexose-accumulating tomato genotype; S, sucrose-accumulating tomato genotype; I, immature stage; II, mature green stage; III, breaker stage; IV, pink stage; V, red ripe stage

SWEET proteins are responsible for transporting monosaccharides and/or disaccharides across membranes following a concentration gradient. The results of quantitative real-time PCR analysis of eight *SlSWEET* genes that are mainly expressed in tomato fruits [22] in the two cherry tomato lines (Fig. 6) showed that *SlSWEET1b*, *SlSWEET7a*, *SlSWEET5b*, *SlSWEET11b* and *SlSWEET14* transcripts were more abundant in the S-type cherry tomato than in the H-type. However, *SlSWEET2a* and *SlSWEET12c* were expressed higher in the early stages of fruit development of the H-type cherry tomato fruit than in the S-type. SlSWEET7a and SlSWEET14 has been thought to transport glucose, fructose and sucrose, and SlSWEET12c has been proved to transport sucrose only, while SlSWEET1b, SlSWEET5b, SlSWEET11b and SlSWEET2a can only transport hexose [18, 23]. The results indicated that the sugar accumulation of the H-type and S-type cherry tomatoes were associated with different SWEET proteins.

**Fig. 7:**
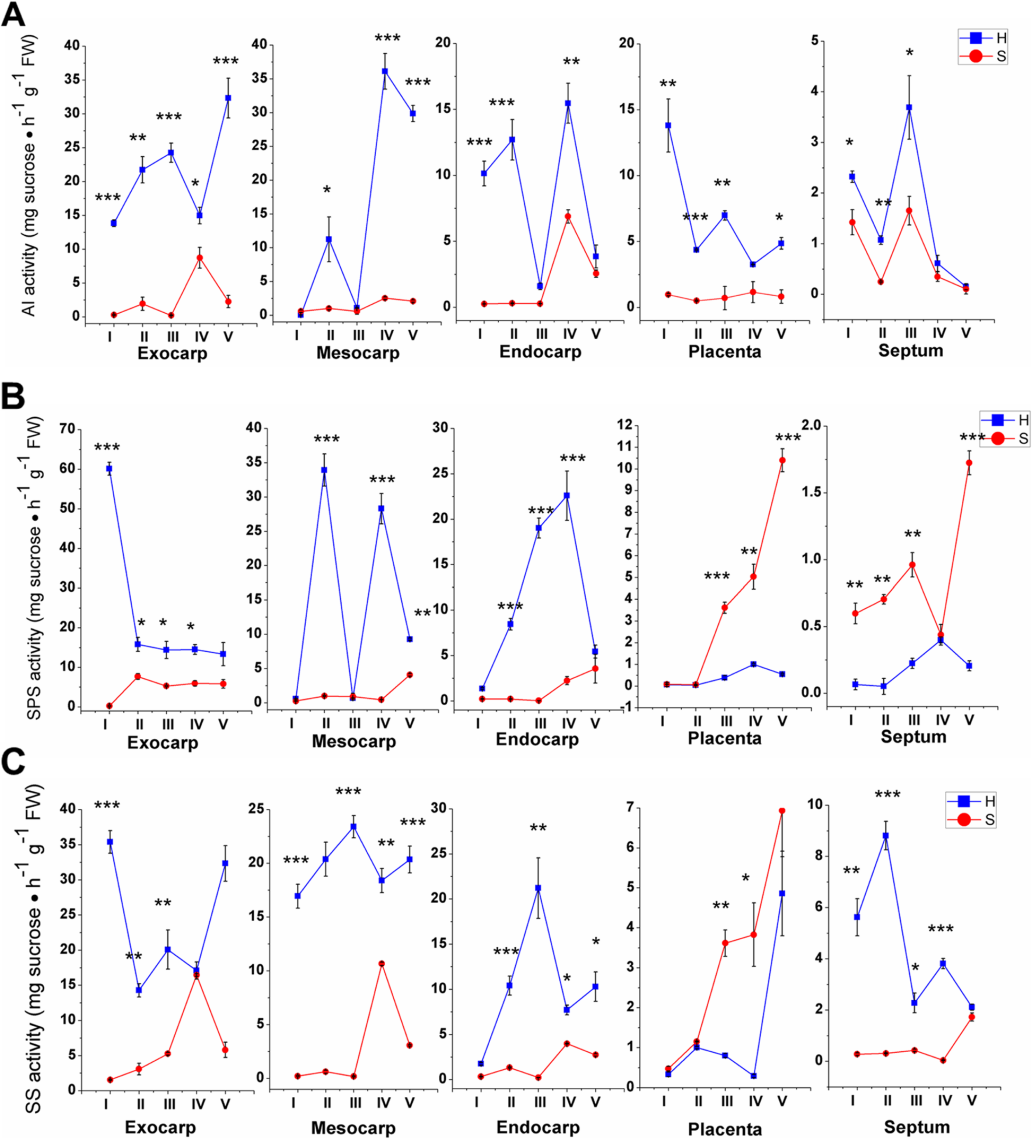
Comparison of the enzyme activities of acid invertase (**A**), sucrose phosphate synthase (**B**), and sucrose synthase (**C**) in different tissues and developmental stages of tomato fruits of different sugar accumulating genotypes**.** Error bars =  ± SEM (n = 3). Student’s t test, *P < *0.05*; **P < *0.01*; ***P < *0.001*. Abbreviations: FW, fresh weight; AI, acid invertase; SPS, sucrose phosphate synthase; SS, sucrose synthase; H, hexose-accumulating tomato genotype; S, sucrose-accumulating tomato genotype

Usually, sucrose is cleaved into hexoses within the fruit by either invertases (AI) or sucrose synthase (SS), and the sucrose resynthesis in sink cells is often associated with sucrose phosphate synthase (SPS). Analysis of the activities of sugar-related metabolic enzymes indicated that the H-type cherry tomato had higher activities of AI activity than the S-type at almost all fruit stages and tissues, and the activity peaked at the pink stage (stage IV), indicating that a large amount of sucrose hydrolysis may occur during this stage (Fig. 7A). The activities of SPS and SS could be detected in both cherry tomato lines, but the activities were higher in the S-type cherry tomato than in the H-type (Fig. 7B), indicating more active hydrolysis and synthesis of sucrose (Fig. 7C).

These results indicated that LeSUT1, SlSWEET2a, SlSWEET12c and AI, likely play key roles in the H-type cherry tomato and are, responsible for sucrose loading into phloem PCs and breaking down sucrose to glucose and fructose. However, LeSUT2, SlSWEET1b, SlSWEET7a, SlSWEET5b, SlSWEET11b, SlSWEET14, SPS and SS possibly play important roles in the S-type tomato, and are responsible for sucrose transport and accumulation and homeostasis of sugar.

## Discussion

### Different types of sugar accumulation in tomato fruits

Sugars are most important factors affecting plant growth and fruit quality [24]. And they are not only important form of stored energy in many plant storage organs but also signaling molecules that form complex regulatory network with other signals such as hormones and nitrogen, and regulate genes expression and plant growth through signal transduction mechanisms [4, 25, 26].

Mature fruits of most ordinary cultivated tomato genotypes accumulate mainly monosaccharides such as glucose and fructose, while high sucrose accumulation has been observed in a few types of wild tomato and cultivated tomato [9, 10, 14, 27]. High sucrose accumulation may be stably inherited, and leading to more valuable genetic variation [12, 13, 28]. Here, the soluble sugar in the different tissues and developmental stages of the fruits of the two cherry tomato lines, that accumulated sucrose and hexose respectively, were determined (Fig. 2). The carbohydrate content showed no obvious difference among various fruit tissues. Sugar accumulation occurred mainly in the later stages of fruit development, and hexose had an absolute predominance in H-type cherry tomato, while the S-type cherry tomato accumulated more sucrose than the H-type tomato. It is worth mentioning that hexose also constituted a certain proportion (approximately 40%) of the total soluble sugar content in the S-type cherry tomato (Fig. 2). The accumulated hexose might come from the hydrolysis of sucrose, and the accumulated sucrose might come from phloem unloading or resynthesis [29].

### The phloem assimilate unloading pathway affects the type of sugar accumulation

In most plant species, sucrose is the major sugar transported through the phloem except for some specific plants, such as those belonging to Rosaceae, Cucurbitaceae and Scrophulariaceae [30, 31]. In tomato fruit, phloem sucrose is unloaded into the surrounding PCs for cell growth or for storage as a nutrient, and the phloem assimilate unloading pathway affects the type of sugar accumulation. Cytological and molecular biological methods are important means of studying the phloem unloading pathway and exploring the mechanisms of fruit sugar accumulation [7, 32, 33].

After long-distance phloem transport, assimilate are unloaded from the SE/CCs through the apoplastic or symplastic pathway. These two pathways can function separately or exist at the same time, and they can also be transformed into each other under certain conditions, which may be related to the development and function of sink organs. Assimilate unloading from the phloem occurs via the apoplastic pathway in several sink organs that accumulate solute sugar at high concentrations, such as those in strawberry [34] and apple [30], because of the lack of plasmodesmata between SE/CCs and surrounding cells. A shift of phloem unloading from the apoplastic to symplastic pathway is involved in tuberization in potato [32]. In ordinary cultivated tomato, the symplastic unloading pathway exists in young fruit storing starch and is converted to the apoplastic unloading pathway in ripe fruit, which mainly accumulate hexose [21]. The assimilate unloading pathway of sucrose-accumulating tomato fruit has not been reported. Here, through cytological observations, we found that a mixed unloading pathway involving the symplastic and apoplastic was involved in the development of fruit in sucrose-accumulating cherry tomato (Fig. 3; Table 1).

### Sugar metabolism- and transport-related proteins affect the type of sugar accumulation

Proteomics is an important technique for studying the function of proteins in organisms. And the iTRAQ method (isobaric tag for relative and absolute quantification) has been widely used in quantitative plant proteomics research [35–37]. In our study, iTRAQ analysis of two type of cherry tomato fruits also provided useful information. In sucrose-accumulating tomato, sugar metabolism and photosynthesis-related proteins were significantly downregulated, while fatty acid metabolism-related proteins were mostly upregulated, which may be related to plant adaptation to the corresponding sugar accumulation mechanism (Fig. 4). Some researchers have proposed that excessively high concentrations of sucrose could lead to a decrease in the expression level of genes related to photosynthesis in plant-derived tissues, while in sink tissues it can increase the expression levels of genes related to plant growth, sucrose hydrolysis and respiration [38, 39]. Among the differential proteins, we easily found that AI was significantly downregulated in sucrose-accumulating tomato (Fig. 4), it is similar to the results reported in some reference that AI expression was very low in sucrose-accumulating wild-type tomato species [16, 29]. AI is generally considered to be located at the cell wall and breaks down sucrose to glucose and fructose in apoplast and SPS is usually known to be a key enzyme for sucrose synthesis. One study showed that the invertase protein also played a role in sucrose-accumulating tomato fruit [29]. It was surprising that we did not find the expression of sugar transporters, when other membrane proteins, such as the aquaporin PIP could be found in the data. We speculated that tomato fruits had too much water resulting in relatively low sugar transporter proteins content, so they could not be detected used this method. Regardless, these proteomic data provided some references for subsequent research.

Many studies on horticultural plants have demonstrated that sugar accumulation is closely related to sugar transporters and sucrose metabolism enzymes. SUTs are responsible for the transmembrane transport of sucrose and play important roles in phloem loading, long-distance transport, and the development of sink organs. Previous study [17] indicated that LeSUT1 expression was high in source leaves and young developing fruits, whereas LeSUT2 expression increased during fruit development. LeSUT1 was characterized as a high-affinity sucrose/proton co-transporter while LeSUT2 was characterized as a low-affinity sucrose/proton co-transporter. Tomato SUT1 and SUT2 proteins were demonstrated to be co-localized in SEs, but not in storage parenchyma cells of fruits, suggesting a direct or indirect role in phloem unloading [40]. These evidence and previous studies suggested LeSUT2 was more thought to function in the retrieval of sucrose leaked from phloem, but whether LeSUT1 transported sucrose in or out of SEs was unclear [41, 42]. However, SUT4 subfamily members have been suggested to be located on the tonoplast and responsible for transporting sucrose into the vacuole in many studies [43]. And tomato LeSUT4 has been proven to be a tonoplast-localized protein [44]. In our study, *LeSUT1* was highly expressed in the H-type tomato fruit, while *LeSUT2* was more highly expressed in the S-type tomato fruit (Fig. 5). Combined with the unloading pathway of phloem assimilates, we thought that LeSUT1 was responsible for the import or export of sucrose from SEs of fruit phloem in the H-type cherry tomato, and LeSUT2 probably function to recycle leaked sucrose from fruit phloem apoplast in the S-type cherry tomato.

SWEET proteins are another class of sugar transporters that can transport monosaccharides and/or disaccharides across membranes following a concentration gradient, and have been shown to function in many essential developmental and physiological processes, including growth and flower, pollen, nectar and seed development [45–47]. Members of clades I and II were suggested to facilitate bidirectional transport of glucose and fructose, while members of clade III were thought to transport of sucrose, and members of clade VI localized to tonoplast [48]. It has been reported that there were 29 SWEET proteins in tomato, a SWEET allele named Fgr^H^ was involved in sugar regulation in leaves and fruits [20], SlSWEET15 was responsible for fruit and seed development [19], SlSWEET7a and SlSWEET14 was thought to transport glucose, fructose and sucrose and influence fruit development, SlSWEET12c was responsible for sucrose transport and early fruit development [18, 23]. Eight tomato *SWEET* genes that expressed highly in tomato [22] were selected to investigate the transcript levels in the two cherry tomatoes in this study. The results indicated that *SlSWEET1b*, *SlSWEET5b*, *SlSWEET11b*, *SlSWEET7a* and *SlSWEET14* were higher expressed in the S-type cherry tomato, while *SlSWEET2a* and *SlSWEET12c* expressed higher in the early stages of fruit development of the H-type cherry tomato (Fig. 6). That mean SlSWEET1b, SlSWEET5b, SlSWEET11b, SlSWEET7a and SlSWEET14 proteins possibly play important roles in the unloading of hexose and sucrose from the phloem of fruit in the S-type cherry tomato, while SlSWEET2a and SlSWEET12c proteins may play similar roles in the S-type cherry tomato.

In addition to invertase, sucrose synthase (SUS or SS) was another important enzyme that converts sucrose into hexoses in plants and has been reported to be involved in the plant development of multiple tissues and organs, such as leaves, fruits and seeds [49–52]. The reaction by which SS breaks down or synthesizes sucrose is reversible, but it is generally believed that SUS plays a role in breaking down sucrose in plants because it requires an acidic environment to breakdown sucrose, while the synthesis of sucrose requires an alkaline environment [53]. In tomato (*Solanum lycopersicum*), six SUS genes have also been identified and SlSUS1 and SlSUS3 may play important roles in tomato fruit development [52]. Work with promoter-GUS fusions has revealed SUS promoter activity in the tomato phloem [54]. Many reports thought that SS may play a critical role in sugar metabolism in some wild sucrose-accumulating tomato fruits [55, 56]. The key enzyme responsible for sucrose synthesis is sucrose phosphate synthase (SPS), which catalyzes the formation of 6-phosphate sucrose from UDP-glucose and 6-phosphate fructose [52, 57]. SPS genes may also be involved in the regulation of flowering time, flower number, pollen germination and fruit development [58, 59]. There were four SPS genes in tomato and SlSPS1 was highly expressed in tomato fruit [57]. In our study, SS and SPS activities were higher in the S-type tomato than in the H-type (Fig. 7). Combined with the cytological evidence, it indicated that SS and SPS probably be related to the maintenance of homeostasis of the sugar concentration in the phloem cells. That was similar to the results of a study on sucrose accumulation in watermelon fruit, the AI activity was significantly lower in genotypes accumulating high levels of sucrose than in those with low sucrose accumulation. Conversely, SS and SPS activities were higher in genotypes with high sucrose accumulation [60].

In addition, in the H-type cherry tomato fruit, AI hydrolyzed the sucrose in the apoplast to hexoses, which were transported into PCs by hexose transporters [8, 61], while in the S-type cherry tomato fruit, hexose and sucrose in the phloem apoplast space might be transported to PCs by hexose transporters and potential sucrose transporters respectively.

### Schematic model of sugar accumulation mechanism in cherry tomato fruits

**Fig. 8:**
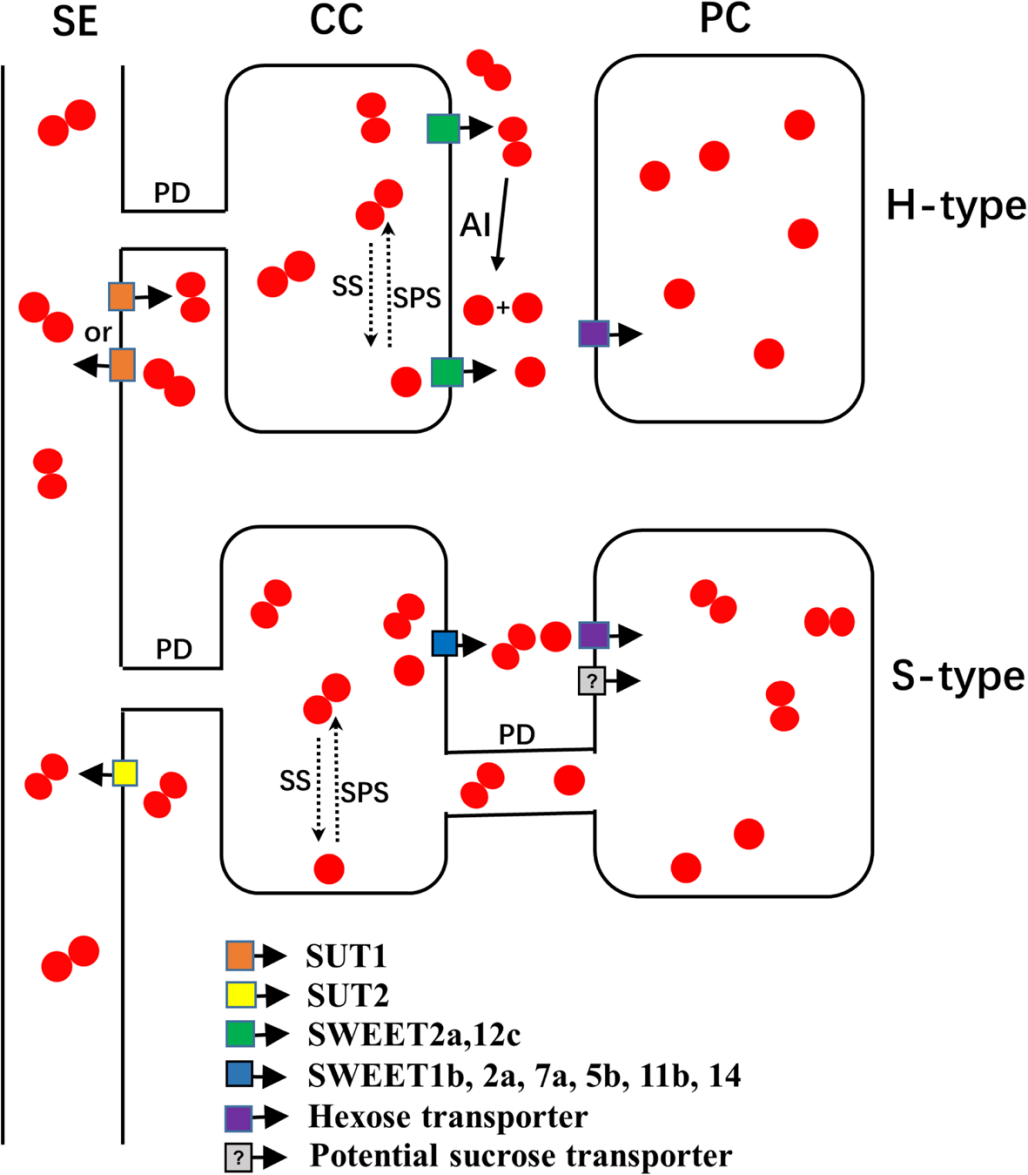
Schematic model of sugar accumulation of two type cherry tomato fruits. Sucrose diffuses into CCs from SEs through plasmodesmata during phloem unloading. In the hexose-accumulating cherry tomato fruit (upper), LeSUT1 located in SEs exports sucrose to the apoplast or retrieves leaked sucrose from the apoplast to SEs is not clear here. Sucrose is transported into the apoplast from SE/CCs maybe by LeSUT1 and SlSWEET12c, and then, it is broken down to glucose and fructose by acid invertase (AI). Small amount of sucrose in SE/CCs is broken down to hexose via SS, and the hexoses are used to resynthesize sucrose under the action of SPS. The hexoses may efflux from SE/CCs by SlSWEET2a. In the sucrose-accumulating cherry tomato fruit (lower), LeSUT2 located in SEs may be responsible for retrieving the leaked sucrose from the phloem apoplast space to SEs. Hydrolysis and synthesis of sucrose via SS and SPS still occur in fruit phloem SE/CCs. The hexose and sucrose are transported to PCs through plasmodesmata, or firstly transported to the apoplast space via SlSWEET1b, SlSWEET5b, SlSWEET11b, SlSWEET7a and SlSWEET14, and then transported into PCs by hexose transporters and potential sucrose transporters. Abbreviations: SE, sieve element; CC, companion cell; PC, phloem parenchyma cell; PD, plasmodesmata; AI, acid invertase; SS, sucrose synthase; SPS, sucrose phosphate synthase; H-type, hexose-accumulating cherry tomato; S-type, sucrose-accumulating cherry tomato

Based on these results we speculated that the phloem unloading pathway for assimilates of hexose-accumulating cherry tomato fruit shifted from symplastic to apoplastic during fruit development [21]. Sucrose was transported into the apoplast from SE/CCs maybe by LeSUT1 and SlSWEET12c, and then, it was broken down to glucose and fructose by acid invertase (AI). Then, these hexoses were transported to PCs via hexose transporters [61]. LeSUT1 located in SEs may also be responsible for retrieving sucrose from the phloem apoplast. Small amount of sucrose in SE/CCs may be broken down to hexose via SS, and the hexose was used to resynthesize sucrose under the action of SPS. The hexose may efflux from SE/CCs by SlSWEET2a. However, during the fruit development of the sucrose-accumulating cherry tomato, a mixed unloading pathway was used. LeSUT2 located in SEs may be responsible for retrieving the leaked sucrose from the phloem apoplast space to SEs. Hydrolysis and synthesis of sucrose via SS and SPS still occur in fruit phloem SE/CCs. The hexose and sucrose were transported to PCs through plasmodesmata, or firstly transported to the apoplast space via SlSWEET1b, SlSWEET5b, SlSWEET11b, SlSWEET7a and SlSWEET14, and then transported into PCs by hexose transporters and potential sucrose transporters (Fig. 8).

## Conclusions

In conclusion, sugar accumulation is a very complex process in tomato, and our research provides detailed evidences from the perspective of cell structure, physiology and molecular biology for elucidation of the tomato sugar accumulation mechanism. These results indicated that AI, SS, SPS, LeSUT1, SlSWEET2a, SlSWEET12c and hexose transporters played more important roles in H-type cherry tomato, while plasmodesmata, LeSUT2, SS, SPS, SlSWEET1b, SlSWEET5b, SlSWEET11b, SlSWEET7a, SlSWEET14, hexose transporters and potential sucrose transporters may play key roles in the S-type cherry tomato. Subcellular localization to fruit phloem cells of key sugar transport and metabolism related genes, and knockout or overexpression of these genes could provide more evidence for this conclusion in future. The study provides a theoretical basis for the improvement of tomato quality and aiding the utilization of tomato genetic resources.

## Methods

### Plant growth

Two stable and homozygous inbred cherry tomato (*Solanum lycopersicum* L. var. *cerasiforme*) lines were used in this research, the sucrose-accumulating type was named ‘TB0023’ (abbreviated as S) and the hexose-accumulating type was named ‘TB0278’ (abbreviated as H). They were supplied by Research Fellow Changbao Li (Beijing Vegetable Research Centre, Beijing Academy of Agriculture and Forestry Sciences) and grown under greenhouse conditions.

### Determination of fruit quality

The vitamin C content was determined by 2,6-dichlorophenol indophenol titration [62]. A 10 g tomato fruit sample was ground into a homogenate and diluted to 100 ml with 2% oxalic acid solution, then filtered and titrated with 2,6-dichlorophenol indophenol. The soluble sugar content was determined by the fluorenone colorimetric method [63]. A 5 g tomato fruit sample was ground into a homogenate and diluted to 100 ml with distilled water, then a certain amount of sample solution is reacted with anthrone sulfuric acid reagent, and then the absorbance is measured at 620 nm, which is converted into the content of soluble sugar. The lycopene content was quantified according to the method of [64]. Methanol and toluene were used to extract lycopene, then the absorbance of the extract was measured at 485 nm with a spectrophotometer. The titratable acid content was determined using the titration method [65]. A 10 g fruit sample was ground in an ice-bath, and distilled water was added to reach a consistent volume of 100 ml. The mixture was filtered, and 2 drops of 1% (m/v) phenolphthalein were added to 20 ml of the filtrate. NaOH was used for titration, and the end point was determined when a pink color appeared and was maintained for 0.5 min.

### Carbohydrate analyses

The analysis of soluble sugars (sucrose, glucose and fructose) was performed by the ethanol extraction method [66] with some modifications. The sample (1 g) was immediately drenched in liquid nitrogen for grinding for 3–5 min. Then, 2–3 ml 80% (v/v) ethanol was added, and the mixture was transferred to a test tube. Then the test tube was placed in a water bath at 80 °C for 1 h. Samples were centrifuged at 4000 g for 10 min, and the supernatants were collected. The pellet was resuspended in 2–3 ml 80% (v/v) ethanol, and three rounds of extraction and centrifugation steps were performed. The three supernatants were combined and evaporated to dryness. Then, the pellet was resuspended in 1 ml of deionized water, filtered through a 0.45 μm filter membrane and analyzed by high-performance liquid chromatography (HPLC). Three biological replicates for each sample and three time repeats for each replicate were conducted for all measurements.

### Fruit cytological observation

Tissue embedding was performed according to a previous method [67]. The vascular bundle and surrounding tissues of fruits at different stages were cut into small pieces of 1–3 mm^3^ by a blade and quickly fixed in 3% (w/v) glutaraldehyde (prepared with 100 mM phosphate buffer solution, pH 7.2). Then pump air until the tissues were completely immersed in the fixative, and fixed overnight at 4 °C. Fixed tissues were washed with the same phosphate buffer (100 mM, pH 7.2), and post-fixed with 1% osmium acid (prepared with 100 mM phosphate buffer, pH 7.2), dehydrated in ethanol, and embedded in Spurr resin. Ultrathin sections were observed under a HITACHI-7650 transmission electron microscope. The measurement of plasmodesmal densities between SE and CC, SE and PC, CC and PC, PC and PC was performed according to a previous method [68].

### qRT-PCR analysis

Total RNA was extracted from 100 to 200 mg of frozen fruit tissue and reverse transcribed into cDNA using an RNA extraction kit (Takara) and reverse transcription kit (Takara) according to the manufacturer’s protocol. Gene-specific primers and internal control (Actin mRNA) primers (Table S1) were used to amplify PCR products on an ABI 7500 system (Bio-Rad). qRT-PCR was performed using SYBR Premix Kit (Takara). Three biological replicates (samples from three individual plants) were performed, and relative amounts of mRNA were calculated using the 2^−△△CT^ method [69].

### Enzyme extraction and activity assays

Enzyme extraction was performed according to [70] and with some modifications. Fruit material samples (1 g) were homogenized in 10 ml 4-(2-hydroxyethyl)-1-pipera-zineethanesulfonic acid (HEPES) buffer (50 mmol∙L^−1^ HEPES–NaOH, 1 mmol∙L^−1^ ethylenediaminetetraacetic acid (EDTA), 10 mmol·L^−1^ magnesium chloride (MgCl_2_), 2.5 mmol·L^−1^ dithiothreitol (DTT), 10 mmol·L^−1^ ascorbic acid (Vc), 5% polyvinylpyrroli-done, pH = 7.5). A small amount of quartz sand was added and ground into a homogenate on ice. The homogenates were centrifuged for 20 min at 12,000 r/min at 4 °C. The supernatant was collected, 5.6 g ammonium sulfate was added, dissolved and the mixture was centrifuged for 30 min at 12,000 r/min at 4 °C. The supernatant was removed, and the precipitate was dissolved in 2–5 ml HEPES buffer. Then the solution was dialyzed with a semipermeable membrane and 10 times diluted HEPES buffer for 20 h.

Acid invertase (AI) activity was enzymatically assayed according to [71]. Sucrose synthase (SS) and sucrose phosphate synthase (SPS) activities were determined as described previously [72].

### iTRAQ-based protein profiling

Protein sample extraction, iTRAQ labeling, and LC–MS/MS analysis were performed according to [73, 74]. The protein content was detected by Bradford method [75]. Ripe fruits of sucrose-accumulating and hexose-accumulating cherry tomatoes were extracted individually and cut into small pieces, immediately frozen in liquid nitrogen and stored at -80 °C for further studies. Five biological repeats were performed, and each measurement was repeated three times. Differential protein analysis, Clusters of Orthologous Groups (COG) analysis and Kyoto Encyclopedia of Genes and Genomes (KEGG) [76] analysis methods were used for data processing.

## Supplementary Information

**Additional file 1:** **Figure S1.** Comparison of plant height (A), leaf number (B), flower number(C) and fruit number (D) between two kinds of tomato genotypes. **Figure S2.** Standards used to define the developmental stages of tomato fruits. **Figure S3.** Kyoto Encyclopedia of Genes and Genomes (KEGG) analysis of differentially expressed proteins involved in starch and sucrose metabolism (A), photosynthesis (B) and fatty acid degradation (C). **Figure S4.** Extraction and quality detection of proteins from tomato fruits for iTRAQ proteome sequencing. **Table S1. **Primers for sugar transporter genes used for qRT-PCR.

**Additional file 2:** **Figure S5.** Full scan of SDS-PAGE gel shown in Figure S4 (B), red frame from left to right displayed the original blots used in the Figure S4 (B).

## Data Availability

The datasets used and/or analyzed during the current study available from the corresponding author on reasonable request.

## Abbreviations

H: Hexose-accumulating cherry tomato
S: Sucrose-accumulating cherry tomato
I: Immature stage
II: Mature green stage
III: Breaker stage
IV: Pink stage
V: Red ripe stage
AI: Acid invertase
SPS: Sucrose phosphate synthase
SS: Sucrose synthase
SUT: Sucrose transporter
SWEET: Sugars will eventually be exported transporter proteins
SE: Sieve element
CC: Companion cell
PC: Phloem parenchyma cell
PD: Plasmodesmata
