# Supplementary material for: Systematic analysis of the sugar accumulation mechanism in sucrose- and hexose- accumulating cherry tomato fruits
Source: BMC Plant Biol. 2022 Jun 22;22:303. doi: 10.1186/s12870-022-03685-8 (PMC9215100; doi:10.1186/s12870-022-03685-8)
Supplement: Supplementary file 1 — Additional file 1: Figure S1. Comparison of plant height (A), leaf number (B), flower number(C) and fruit number (D) between two kinds of tomato genotypes. Figure S2. Standards used to define the developmental stages of tomato fruits. Figure S3. Kyoto Encyclopedia of Genes and Genomes (KEGG) analysis of differentially expressed proteins involved in starch and sucrose metabolism (A), photosynthesis (B) and fatty acid degradation (C). Figure S4. Extraction and quality detection of proteins from tomato fruits for iTRAQ proteome sequencing. Table S1. Primers for sugar transporter genes used for qRT-PCR. [file 12870_2022_3685_MOESM1_ESM.docx]

**Supplementary information**


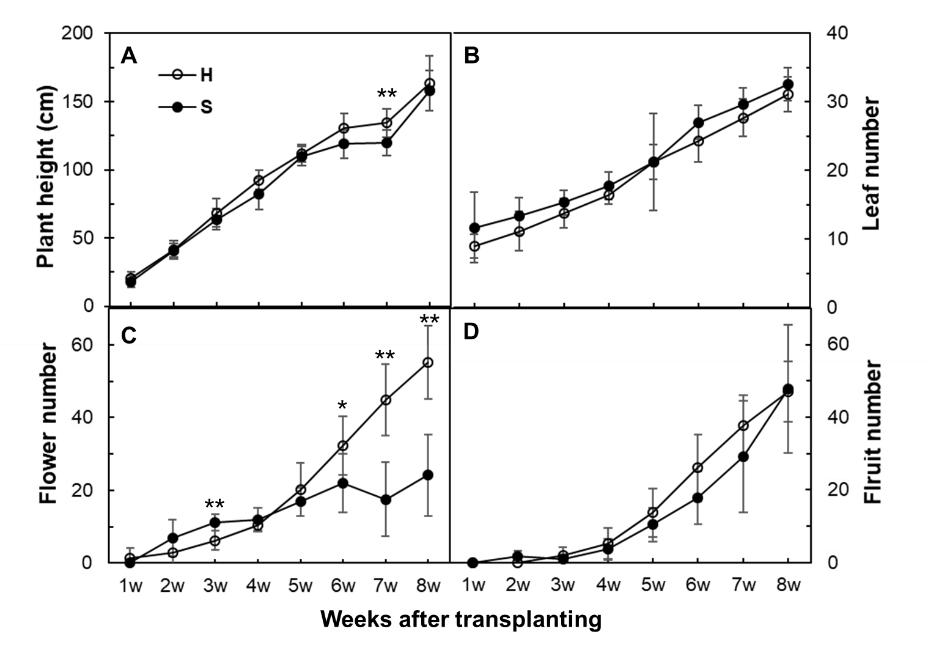


**Figure.** **S1** Comparison of plant height (A), leaf number (B), flower number(C) and fruit number (D) between two kinds of tomato genotypes. Student’s t test, *P < 0.05; **P < 0.01, n=9. Abbreviations: H, hexose-accumulating cherry tomato; S, sucrose-accumulating cherry tomato.

**
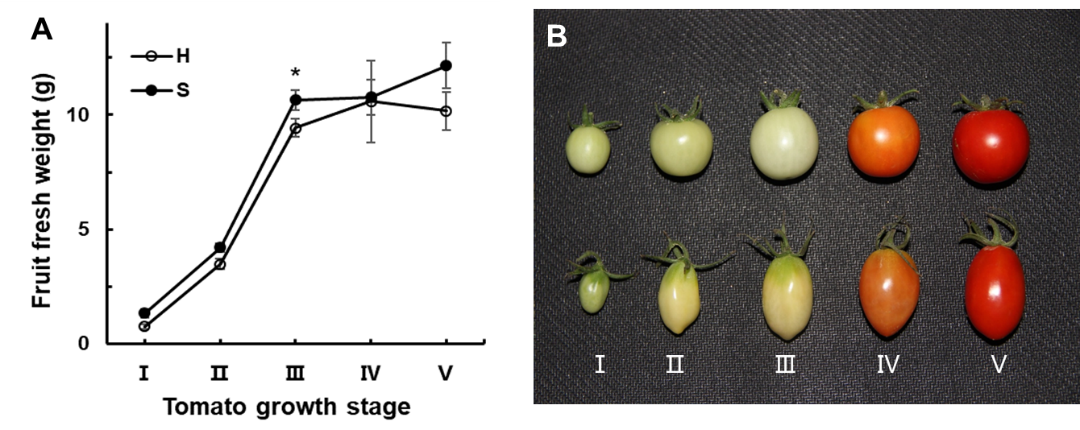
**

**Figure.** **S2** Standards used to define the developmental stages of tomato fruits.

A, Growth curve of tomato fruit. Student’s t test, **P < 0.05*, n=5. B, Five developmental stages of fruits of two cherry tomato species based on United States Department of Agriculture (USDA) standards. Abbreviations: I, Immature stage; II, Mature Green stage; III, Breaker stage; IV, Pink stage; Ⅴ, Red Ripe stage. H, hexose-accumulating cherry tomato; S, sucrose-accumulating cherry tomato.

**
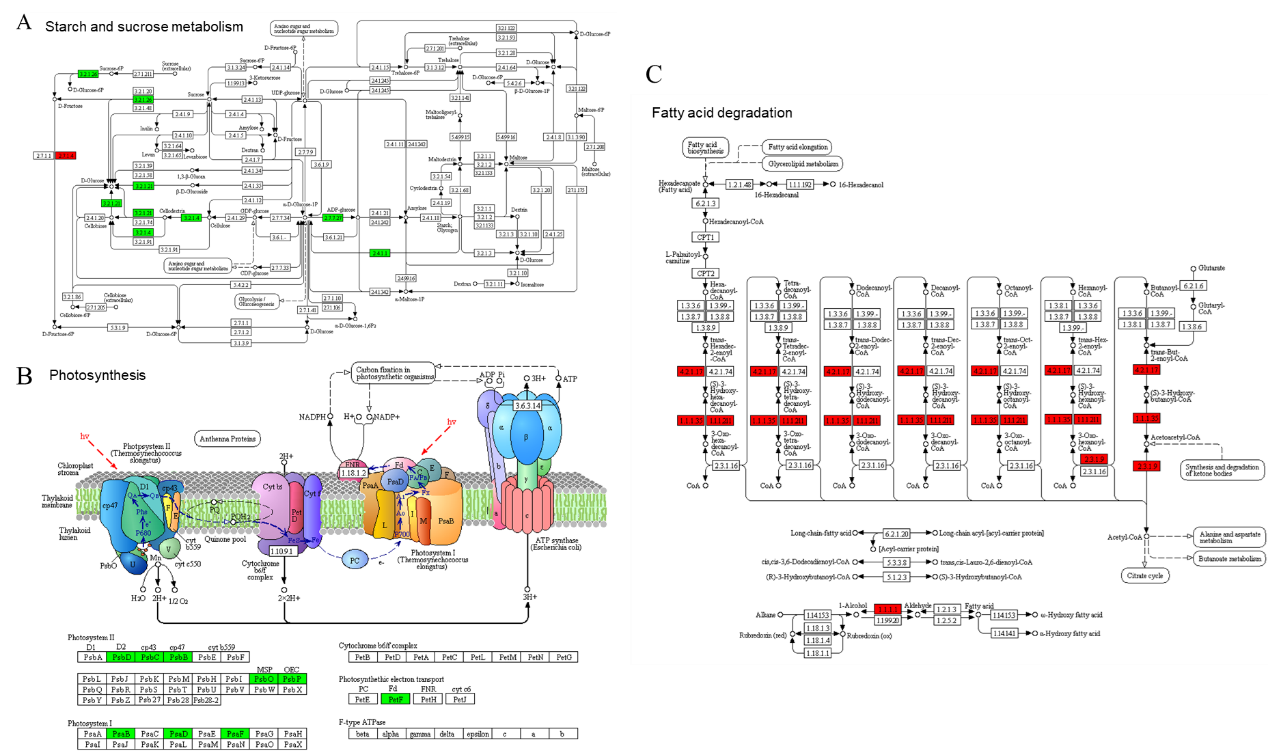
**

**Figure.** **S3** Kyoto Encyclopedia of Genes and Genomes (KEGG) analysis of differentially expressed proteins involved in starch and sucrose metabolism (A), photosynthesis (B) and fatty acid degradation (C). The green box represents downregulated proteins and the red box represents upregulated proteins. KEGG analysis was performed through www.kegg.jp/kegg/kegg1.html.

**
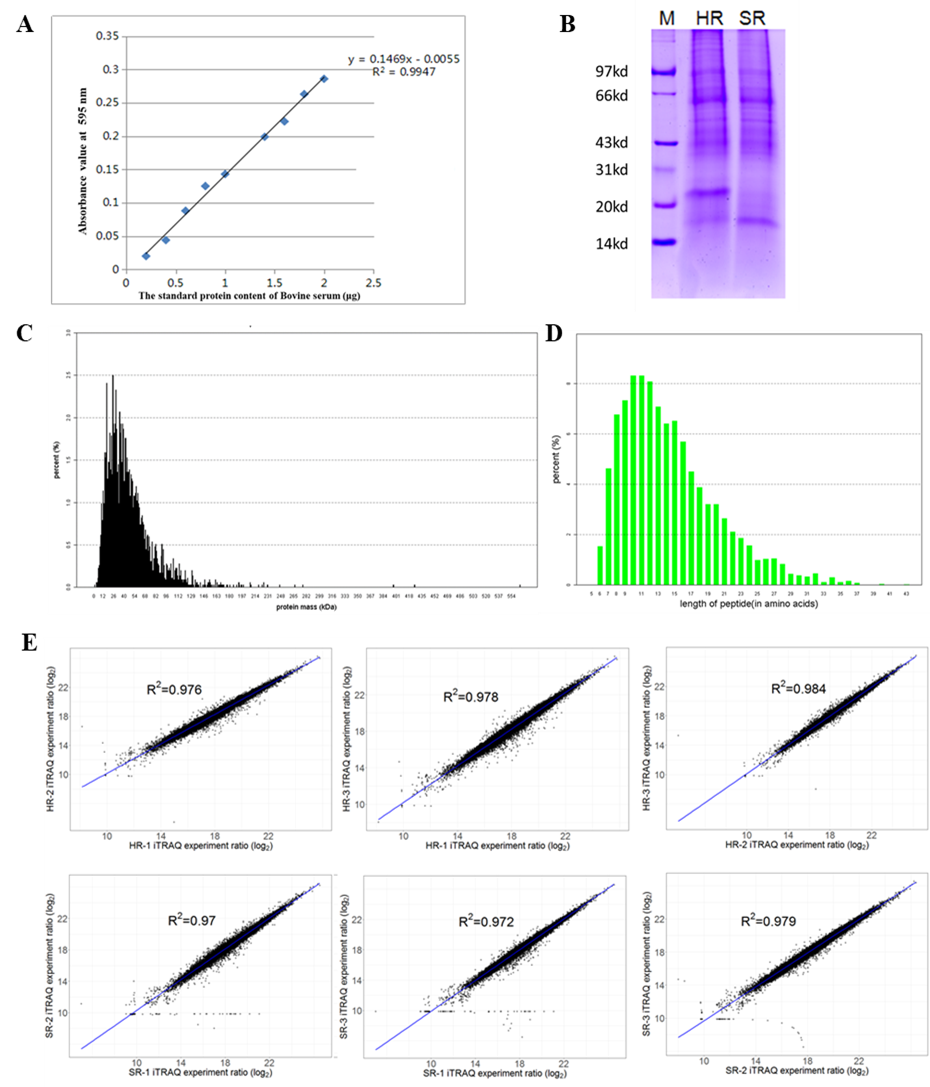
**

**Figure.** **S4** Extraction and quality detection of proteins from tomato fruits for iTRAQ proteome sequencing. A, The standard protein content of bovine serum; B, Protein quality detection by SDS-PAGE，the gel image has been cropped for improving the clarity and conciseness of the presentation, original blots/gels are presented in Supplementary Figure S5; C, The distribution of protein mass; D, The distribution of peptide length; E, Parallel analysis between samples.

**Table S1.** Primers for sugar transporter genes used for qRT-PCR.

| **Gene** | **ID** | **Forward Primer(5’-3’)** | **Reverse Primer(5’-3’)** |
| --- | --- | --- | --- |
| *LeSUT1* | NM_001302901.2 | ACCACGAGCGATTGCTGTAT | CAGCCAGTAGTGCTCTGCAT |
| *LeSUT2* | [NM_001247392.2](https://www.ncbi.nlm.nih.gov/entrez/viewer.fcgi?db=nucleotide&id=926234465) | AAGGAGAAGCAGATGAAGTAA | CTAACGCCAAGAACAACAG |
| *LeSUT4* | [NM_001247415.2](https://www.ncbi.nlm.nih.gov/entrez/viewer.fcgi?db=nucleotide&id=909618044) | CATAGAACAAGGCATAAC | CTCGTAACAATAGTCTCA |
| *SlSWEET1b* | Solyc04g064620 | ATCTTCTCAACTGTTGCATTGG | AGGACAAGAAGAAAGGCATGTA |
| *SlSWEET1c* | Solyc04g064630 | TCTACCTATGCACCATTTTGCT | ATACCAAGCTGAAAACAAGCAG |
| *SlSWEET2a* | Solyc02g071520 | GAATGTCAGTTGTAGGTGGACT | AATACCAGGAGACACAATTGGT |
| *SlSWEET5b* | Solyc06g071400 | CGGATTTGGTCTTGCTATTGAG | TGAAGACTATAGCCACAATCCC |
| *SlSWEET5b* | Solyc08g082770 | CATCTTGTTCTTGTCACCCTTG | CCCAAAGCCCACAATTGATAAA |
| *SlSWEET7a* | Solyc08g082770 | CATCTTGTTCTTGTCACCCTTG | CCCAAAGCCCACAATTGATAAA |
| *SlSWEET11b* | Solyc03g097570 | ATAGCATCCTTGGGACCTTTAG | GAGAAGTGTTGTGTTGGTCTTC |
| *SlSWEET12c* | Solyc05g024260 | AGTCTCCATCAATTCCTTTGGT | AATCCAACCAACAACTTGTACG |
| *SlSWEET14* | Solyc03g097560 | AACATTGTCTCCTTCATGGTCT | AAAGCATGGCACTGAATAATCC |
| Actin-7 | NC_015440.2 | GGAATGGGACAGAAGGAT | CAGTCAGGAGAACAGGGT |
