# Supplementary material for: Systematic analysis of the sugar accumulation mechanism in sucrose- and hexose- accumulating cherry tomato fruits
Source: BMC Plant Biol. 2022 Jun 22;22:303. doi: 10.1186/s12870-022-03685-8 (PMC9215100; doi:10.1186/s12870-022-03685-8)
Supplement: Supplementary file 2 — Additional file 2: Figure S5. Full scan of SDS-PAGE gel shown in Figure S4 (B), red frame from left to right displayed the original blots used in the Figure S4 (B). [file 12870_2022_3685_MOESM2_ESM.docx]

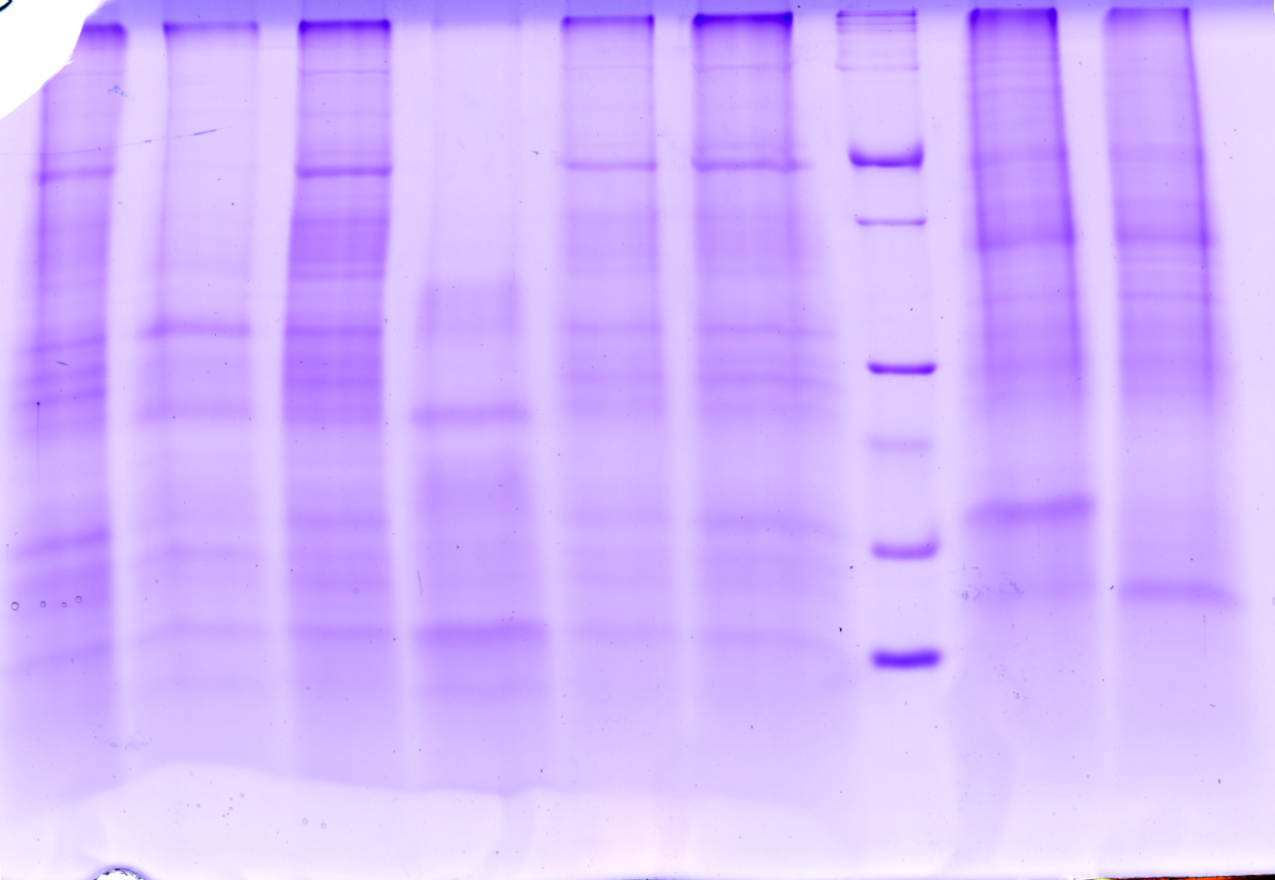


**Figure. S5** Full scan of SDS-PAGE gel shown in Figure S4 (B), red frame from left to right displayed the original blots used in the Figure S4 (B).
